# Function of Perceived Corporate Social Responsibility in Safety of Sports Activities and Home Aerobic Equipment in the Late Period of COVID-19

**DOI:** 10.3389/fpsyg.2022.919254

**Published:** 2022-06-20

**Authors:** Lang Ma, Jiang Liu, Yicheng Liu, Yue Zhang, Chunmei Yang

**Affiliations:** ^1^Physical Education Department, Southwest Jiaotong University, Chengdu, China; ^2^Intensive Care Unit, Neijiang Hospital of Traditional Chinese Medicine, Neijiang, China

**Keywords:** perceived CSR, health up-gradation, sports safety measures, injury risk perception, injury prevention expectation

## Abstract

The pandemic has impacted various industries, including the sports industry. However, corporate social responsibility (CSR) can mitigate the adverse effects of the crisis and promote the sports industry. To analyze the effect of CSR, the study examined the impact of perceived corporate social responsibility on injury prevention expectation, injury risk perception, and health up-gradation with the mediation of sports safety measures. There are 259 sportsmen of local sports bodies provided the data through a self-administered survey. Data analysis was conducted through Smart-PLS and SEM techniques. The outcome of the analysis showed that perceived corporate social responsibility leads to injury prevention expectation, injury risk perception, and health up-gradation. Also, the study found that sports safety measure mediates the relationship between perceived corporate social responsibility and injury prevention expectation, between perceived corporate social responsibility and injury risk perception, and between perceived corporate social responsibility and health up-gradation among sportsmen of local sports bodies. The theoretical implications were presented related to the significance of CSR and sports safety measure and their impact on sportsmen injury prevention expectation, health, and risk perception. The practical implications were related to the management of local sports bodies and how they can induce CSR initiatives and programs. Some limitations related to sample size, incorporating other variables, examining the model in other contexts, and using different study designs, have also been mentioned in the study.

## Introduction

Regular physical activities including sports has not been remain the same during pandemic. Such unexpected changes in activities lead to behavioral modifications of peoples (Dwyer et al., 2020; Woods et al., 2020; Polero et al., 2021). According to Ammar et al. (2020), lockdown during COVID-19 had an impact on public. For example, reduction in physical activity levels, rise in daily sedentary behavior, and an increase in unhealthy dietary patterns were seen. Some other researchers also got similar results (Oliveira et al., 2021). These sudden developments impacted everyone. The people pursuing their physical activities in gyms, grounds, and other locations were severely impacted. People were compelled to stay at homes because sports facilities and public parks were closed. Closure of such public places disrupted normal sports activities and affected their aerobic workouts.

The people were forced to stay at home for a longer period of time. It became difficult for them to maintain their fitness levels. They had to go through the experience of limited physical activities, limited socialization, insecurity, and hopelessness. All these restrictions can contribute to cognitive and emotional health problems (Ammar et al., 2020). Researchers discovered that adults experience psychological problems when they change their existing lifestyles in response to the spread of a disease. However, it is also observed that strong coping skills, abilities, and physical exercises are beneficial in managing such health-related issues (Chtourou et al., 2020). People can maintain their fitness through athletics and other sports activities. Such activities are useful in maintaining health status. These also assist in fighting the detrimental consequences of high bp, heart disease, and lung ailments. During the early days of COVID-19 spread, several health-related constraints were seen on rise. This rise was observed majorly due to lack of physical activities. According to researchers, lack of physical activities contributes to shortening of life expectancy and numerous physiological health disorders (Bentlage et al., 2020). Several pulmonary, cardiovascular, neuromuscular, neurological, and skeleton systems are associated with normal body functioning. Sports activities play an important role in regulating these processes. Some of the body processes are associated with maintenance mechanisms (Nawaz et al., 2020b). These include hormone release, gastrointestinal, immunological, and kidney processes. These processes are vital in combating real or fictional risks to the body. All these processes are rectified when individuals get involved in sports activities. Hence, physical exertion and some other precautions are useful in combating the COVID-19 (Chen et al., 2020).

According to health experts, regular exercise may reduce the chances of respiratory failure. It has been one of the leading causes of mortality in patients of COVID-19. There is enough evidence suggesting that athletics can contribute to promoting mental well-being (Mazyarkin et al., 2019). It has become extremely hard for some people to achieve the basic criteria of healthy lifestyle given by WHO during COVID-19. The condition is getting worse, as sports and daily workouts are not accessible at these times (Bentlage et al., 2020). There has been a crucial concern of health for people in the wake of restrictions during disease outbreak. They have to survive in these times and seek ways to handle their fitness levels. During home quarantine and locked gymnasiums, playgrounds, and exercise facilities, it is becoming difficult (Hao et al., 2020; Nawaz et al., 2020a). It has been observed that sports activities carry along some of the risks. These risks are associated with sports every time whether on normal days or during pandemic times (King et al., 2020; Lim and Pranata, 2021; Senişik et al., 2021). Outdoors sports activities and aerobic activities at home increase respiration rates. While in times of pandemic, it is advised by WHO that those activities which contribute to elevating the respiration rate, must be administered watchfully to avoid any transmission of coronavirus. Therefore, this study tried to explore the risks attached with sports activities, safety measures, injury prevention expectations, injury risk perception and health up-gradation. Moreover, the most important role of perceived corporate social responsibilities (CSR) was also the main aim of the study. For example, immunization has been a greater tool of safety during sports since the invention of COVID-19 vaccines. Sports organizations paid good attention to this aspect of safety of the sportsmen.

Insufficient immunization rates were considered as a major public health issue in the developed countries like USA and around the world. Despite the fact that safety measures for sportspersons in amateur sports have received a lot of attention, immunization has been largely ignored in these talks. However, it is considered that sportsmen, viewers and professionals such as referees at recreational sports events are equally at risk of contracting dangerous infections. During early spread of COVID-19, Olympic games to be held in 2020 in Japan were suspended due to the possible spread of disease. It also impacted other professional and amateur sporting events around the world and caused postponement or rescheduling (Francis and Francis, 2020). To overcome these losses, immunization of athletes and participants became a high priority as a safety measure during late times of COVID-19. Sport and recreation participation is crucial for the current and future public health benefits of young population. Psychosocial advantages, increased self-esteem, physical skills development, interpersonal skills, cooperation, competitiveness, reducing stress, and general wellness are all advantages of continued sport engagement in youth. Adult participation rates in sports activities are declining. Sport and physical injury may be contributing to this strain. According to reports, annually 8 % dropouts from sports are due to injury (Emery, 2018). This high rate of injury emphasizes a need for safety measures to lower the risk of injury, prevention from injuries and health up gradation. There is also a need to research the importance of organized team sports in injury epidemiological literature (Emery, 2018).

Sports participation may be hampered by minimizing the danger of injury. Sporting activities and sports clubs offer chances and environments for people to be engaged and lead a healthy life. This seems to be a prominent approach considering that physical inactivity is a major health concern right now (Cao et al., 2018). Lower-limb injury accounts for more than 60% of the recreational sports injury burden. While concussion is still a hot topic in youth sports, accounting for 15% of the injury load. Decreased physical activity, increased obesity, post-traumatic arthritis, and post-concussion disorders are all possible consequences of sports injuries (Whittaker et al., 2015). Physical activity involvement is a predictive factor of numerous incidences of deaths (Whittaker et al., 2015). The quality of life can be improved through the encouragement of physical activity and lowering the risk of all sports injury. This is especially required in adventurous and action sports. Injury risk can be investigated after the level of injury in a target group has been determined through surveillance. Following the identification of risk factors, an injury prevention approach is developed and validated. This is done through evaluation of the preventive approach utilizing suitable surveillance (Emery, 2018). In recent years, professional sports have prioritized the operational backdrop for injury prevention. Professional sports organizations are trying their best to provide promising practices and policies (Verhagen et al., 2010).

There is a need for cost-benefit analysis while devising policies for injury prevention plans. Such assessments can benefit the process of providing injury prevention measures in sport activities, particularly adventurous sports (Marshall et al., 2016). Currently no studies have gone through injury prevention expectations, injury risk perception and health up gradation of sportsmen in a single framework which showed a significant gap in management research. Moreover, at organizational level, no such resources were provided to the sportsmen with responsibility. Therefore, this research tries to fill the gap by evaluating role of perceived corporate social responsibility on sports safety measures, risk of injury expectations, injury prevention and health up gradation of sportsmen. The global pandemic has a profound impact on public life and sports in a ways that have never been seen previously. The epidemic and lockdowns are an unanticipated external disruption to the sport system. Therefore, sport organizations have to make standard operating procedures every time before proceeding toward tournaments and activities. On one hand, organizations require income from their primary business of sporting events and contests. On the other hand, they also have a financial obligation toward their suppliers and partners (Thormann and Wicker, 2021). The social role of these sports organizations argues that they should act in a socially responsible way. During pandemic, one of the expressions frequently used by sport authorities was “sport's societal responsibility.”

The phrase is defined in policy statements as an essential component of social life that brings people from many cultural origins together. It expresses societal fundamental elements, and promotes public discussion and inclusiveness (Thormann and Wicker, 2021). Corporate social responsibility (CSR) is commonly used to investigate societal responsibilities. The German Football League is a legally recognized organization. This organization applies CSR principles in sports in the very same manner as they do with certain other businesses. Sport regulatory agencies are subject to CSR standards because they are legally organized non-profit organizations that follow the laws of their respective nations (Chelladurai, 2016). Previous research has presented various examples of CSR operation in non-profit sport organizations. The findings emphasized the necessity of CSR in these organizations to project a favorable image, acquire taxpayer support, and improve the friendliness of their primary stakeholders. The pandemic provides a unique opportunity for sport organizations to practice CSR. CSR initiatives may focus on demonstrating overall obligation more than an organization's financial liabilities (Thormann and Wicker, 2021). Keeping in view the significance of perceived CSR in sports, this research tries to find out the role of CSR in sports safety systems during late times of COVID-19 which was not studied ever before.

## Theoretical Framework

This research gets support from stakeholder theory (Freeman, 1984), which is already in practice by many sports organizations. In order to claim extraordinary standards for themselves, sport organizations rely on significant acceptance from the public (Varmus et al., 2018). Any organization or individual who has a role in achieving the goals of a company is regarded as a stakeholder. There may be some other external stakeholders of sports organizations like supporters and fans. It is difficult to evaluate all these stakeholders through questionnaires. Rather, qualitative approach is suitable and viable to record the viewpoints of them. The variability and size of population suggests that huge number of participants may be evaluated through quantitative approach, and it is useful (Thormann and Wicker, 2021). The stakeholder theory established by (Freeman, 1984) is used in several CSR conceptualizations. Rather than focusing solely on revenue generation for the sake of shareholders, this theory recognizes that firms are accountable and may generate value for a wide range of stakeholders (Freeman, 1984). As a result, some academics emphasized firms' responsibilities for their customers, employees, authorities, subsequent generations, global ecosystems, and community as a whole (Pérez and Rodríguez del Bosque, 2013). Moreover, one of the primary reasons for sports teams and organizations to embrace CSR practices is pressure from external stakeholders. It highlights the significance of the perception of CSR among stakeholders (Kolyperas et al., 2015).

This conceptual basis is consistent with Carroll's original view of CSR, which is multifaceted in design and incorporates aspects of economic, regulatory, moral, and voluntary duties (Carroll, 1979, 1991). The responsibility of a corporation to produce valuable items for consumers at a reasonable cost, while remaining successful and generating wealth for shareholders, is known as economic stewardship. A regulatory aspect relates to a company's need to follow the law and adhere to legal provisions in its activities. Moral behavior extends beyond society's normative expectations. Such behavior provides an adherence to ethical norms even if they are not necessarily governed by legislation. According to Carroll (1979) and Carroll (1991), organizations achieve legitimacy by adhering to ethically correct societal characteristics. Finally, voluntary responsibility encompasses altruistic or charitable activity aimed at the well-being and growth of society. Carroll's multifaceted CSR conception seems represented in the European Commission's definition as well. CSR refers to firms having obligations and acting in ways that go beyond their legal requirements and corporate objectives. Such broader obligations encompass a broad range of topics, and they are typically summed up as social and environmental ones. The notion of social refers to community as a whole instead of only social policy matters (Thormann and Wicker, 2021).

Scholars have begun to evaluate the emergence of CSR within current strategic policies. These investigations are built upon requests for inquiry as to how things work when CSR is introduced in a company (Mirvis and Googins, 2007). Previously, internal and external determinants of social change in an enterprise, as well as the structure of CSR programs and policies have been studied (Russo and Tencati, 2009). However, in sports, a field of CSR which may deal with cooperation, competition and cooperative competition is still developing. Because competitive sport is both a source of direct and indirect competition. This competition may arise between teams and leagues, sports and other forms of entertainment, sponsors and fans. Such competition may be a source of cooperation as well. The institutionalization of CSR may vary across the organizations (Kolyperas et al., 2015).

This study also gets support from social exchange theory and social identity theory which focus on performance of sportsmen (Back, 1965). If they are given socially responsible safety measures during the course of sports activities, then in exchange they may perform better in sports. So, the provision of safety measures during sports activities works exchange-ably with the efforts put in by the sports organizations or teams. Similarly, drawing upon social identity theory, CSR positively predicts the performance of the players, or the ones involved in sports activity (Askarian and Raahbar, 2021). Injury prevention expectation, injury risk perception and health up gradation are all possible outcomes of positive outcomes of perception of CSR in directing the safety measures in sports activities. Therefore, this study is strongly supported by stakeholders' theory, social exchange and social identity theory.

### Perceived CSR, Sports Safety Measures, Injury Prevention, Injury Perception and Health

Sport organizations have historically been known for their capacity to deliver social benefits such as better physical health, improved education, and social integration (Jarvie, 2003; Myers et al., 2004; Lambourne, 2006). The idea of CSR has exploded in popularity all across major sports sectors. It's becoming more common in professional sports, including an expanding list forming partnerships, economic, and societal entities in order to effectively engage with a variety of stakeholders. CSR perception in sporting settings, particularly clubs, is important for managers and sports management staff. Because sport companies differ from one another and from other enterprises of main activities, organizational structures, mindsets, and strategies (Dar et al., 2022).

It is important to understand how CSR activity unfolds, develops, and evolves within such unusual and socially created organizations. It is also useful to explore causes, obstacles, and organizational stages to develop additional CSR perceptions about athletics as well as other domains of sports (Kolyperas et al., 2015). Several studies on sport organizations' CSR have been undertaken in a variety of situations such as professional team sports, sport competitions, and sport federations (Babiak and Wolfe, 2006; Walters and Tacon, 2013; Walzel et al., 2018). Prior study on CSR activities focused on community-based activities with an emphasis on sports activities, education of youth, and their development. Additional actions that can be classified as corporate giving for philanthropic purposes include gifts, ticket freebies, and fundraising campaigns (Inoue et al., 2017; Rowe et al., 2019). This focus on the improvised citizen perception which sees CSR as an instrument for achieving organizational objectives is unusual. Babiak and Wolfe (2006) ended up choosing to probe the morally acceptable and voluntary dimensions of CSR as fulfilled by consensual, implicit actions in their first study. Various factors which encourage organizations to engage in CSR activities were previously investigated. These include external influence from other stakeholders such as competing clubs and internal operations which push professional sports teams' CSR efforts (Kolyperas et al., 2015). Financial autonomy and human resources are two major motivators for sports federations. The lack of such resources is also one of the most significant barriers to developing formal CSR programs (Zeimers et al., 2021).

Researchers have used many techniques to explore CSR in sporting (Lacey and Kennett-Hensel, 2016; Thormann and Wicker, 2021). The results of previous studies used qualitative research methodologies. Qualitative studies were chosen, particularly in professional team sports, with data acquired through observations and interviews, focus groups, and experimental research. The majority of qualitative research was done as case studies of various teams, tournaments, and sports organizations (Babiak and Wolfe, 2006; Banda and Gultresa, 2015; Douvis et al., 2015; Babiak and Kihl, 2018; Walzel et al., 2018). Few investigators utilized a mixed methods approach to address their questions of the study with questionnaires being the most common (Davies and Moyo, 2017). Surveys were used to conduct specialized quantitative research. Mostly, these surveys utilized just brief three to five item measures to assess CSR or perception of CSR, which only scratched the surface of CSR's multifaceted character (Lacey and Kennett-Hensel, 2016). Walters and Tacon (2013) made an exception by statistically measuring CSR while maintaining the concept's comprehensiveness. All this literature supported the idea of perceived CSR in sports organizations. Given the popularity of sports activities among sportspersons, most of the existing research on sports-related injuries focuses on elite players or professionals. Physiological changes in both groups, such as muscular strength, physical growth, and variations in combined biomechanics, skill levels, and workloads, the injury landscape, injury risk factors, and injury processes may differ between children and adulthood (Perera et al., 2019).

Seemingly, the findings pertaining to grown-up sportspersons cannot always be applied to younger players. Sports participation may be hampered by the danger of injury. Sports and sporting organizations offer chances and environments for people to be active and lead a healthy life, which is especially significant considering that idleness is a major public health issue right now (Cao et al., 2018). Previously, no researcher tried to find out the role of perceived CSR on sports safety measures, injury risk related expectations, injury risk perception and health up-gradation scope of individuals involved in sports activities both outdoors and home-based aerobic activities. The only study conducted on injury prevention expectations, injury risk perception and problems related to health, was in the field of floorball sport (Perera et al., 2019). This research motivated the authors to conduct the current research. It suggested the possible associations between perceived CSR and these dimensions of injury panorama. Therefore, the following were suggested.

***H***_**1**_***:*** *Perceived corporate social responsibility leads to injury prevention expectation*
***H***_**2**_***:*** *Perceived corporate social responsibility leads to sports safety measures*
***H***_**3**_***:*** *Perceived corporate social responsibility leads to injury risk perception*
***H***_**4**_***:*** *Perceived corporate social responsibility leads to health up gradation*

### Sports Safety Measures

Within the sports management and public health areas, the debate over what duties sports organizations have toward society is gaining traction. Internationally, sports organizations' social responsibility in relation to health improvement strategies and practices has been explored (Kokko et al., 2014). However, little research has been done on how managerial ideals of social responsibility and health up-graduation might be integrated inside sports groups. Participation in group sport adds significantly to recreational physical exercise at health-up gradation levels. Community sports clubs are crucial venues for overall physical activity. Sport is a widely used form of recreational physical activity, particularly amongst children and teens. Additionally, involvement in group sports can have a significant impact on not only physiological but also mental and social health. Because of the communal element of group sports, the social and mental health advantages can be greater than those obtained from participating in individual types of sports activity. Setting-based health up gradation emphasizes pretty much on the entire thinking to handle a variety of behavioral change tactics which promote sports engagement as a healthy habit. It can lead to encouraging people to participate in sports. Interpersonal level elements such as enhancing competence and ability, intrapersonal techniques such as better coaching practices, and physical environmental and policy level effects such as boosting organizational governance and management are all examples of these strategies (Kokko et al., 2014).

In sport organizations around the world, a variety of health promotion methods and policies are being applied. Sport injury prevention, cigarette free locations, appropriate alcohol consumption, sunscreen, eating healthy, drinking healthy beverages, and friendly environments are just a few of them (Kokko et al., 2016). Some are more focused on governmental level. Such strategies and tactics are sometimes led by the organizations themselves or by external health promotion organizations. Although the impact of environmental framework depicts sports injury mechanisms and connects these to the effects and environmental intervention areas. It lacks precise recommendations for developing and implementing practical safety promotional strategies. Contemporary health and safety promotion strategies are built on community mobilization by gathering together relevant agencies and specialists to gather and analyze the data on the injury crisis, and then distributing the results to organizations with the means and authority to take preventive action (Norris and Pittman, 2000). There are a number of factors to consider when addressing sports safety management from a community standpoint. Some athletes, for instance, are members of groups that transcend geographical bounds. They form sports societies which can concentrate on participation in a regional sports organization (Timpka et al., 2006).

If contemporary society health and safety promotion strategies are to be shown in the wide variety of sports, program designs must include making allies with all these global and local sports community members at all tiers. These strategies work well by collecting and processing detailed information through the findings for strategic planning in the pertinent organizational setting. Nonetheless, sporting organizations alone are unable to address all concerns relating to sports safety such as challenges with inadequate sporting venues and drug and alcohol addiction. In order to build safe local services and settings for physical activities, a community-based sport injury prevention program must form partnerships with geographically specified groups (Ross et al., 2021). Currently, no study has found any significant contribution of sports safety measures due to a lack of research. Although, this has a lot of scope to be explored. Therefore, this study tries to find out the mediating as well as direct roles of sports activities and injuries in terms of prevention expectations, injury perception and health up-gradation of people during the late pandemic of COVID-19. This study utilized outdoor sporting activities and home-based aerobic physical activities to be monitored. The above literature in support of sports safety measures also indicated that the proposed role of sports safety measures can mediate between perceived CSR, injury prevention expectation, injury perception and health up-gradation. Therefore, the author tried to find out the possible associations between them through these hypothesis.

***H***_**5**_***:*** *Sports safety measures lead to injury prevention expectation*
***H***_**6**_***:*** *Sports safety measures lead to injury risk perception*
***H***_**7**_***:*** *Sports safety measures lead to health up gradation*
***H***_**8**_***:*** *Sports safety measures mediate the relationship of perceived CSR and injury prevention expectation*
***H***_**9**_***:*** *Sports safety measures mediate the relationship of perceived CSR and injury risk perception*
***H***_**10**_***:*** *Sports safety measures mediate the relationship of perceived CSR and health up gradation*

**Figure 1:**
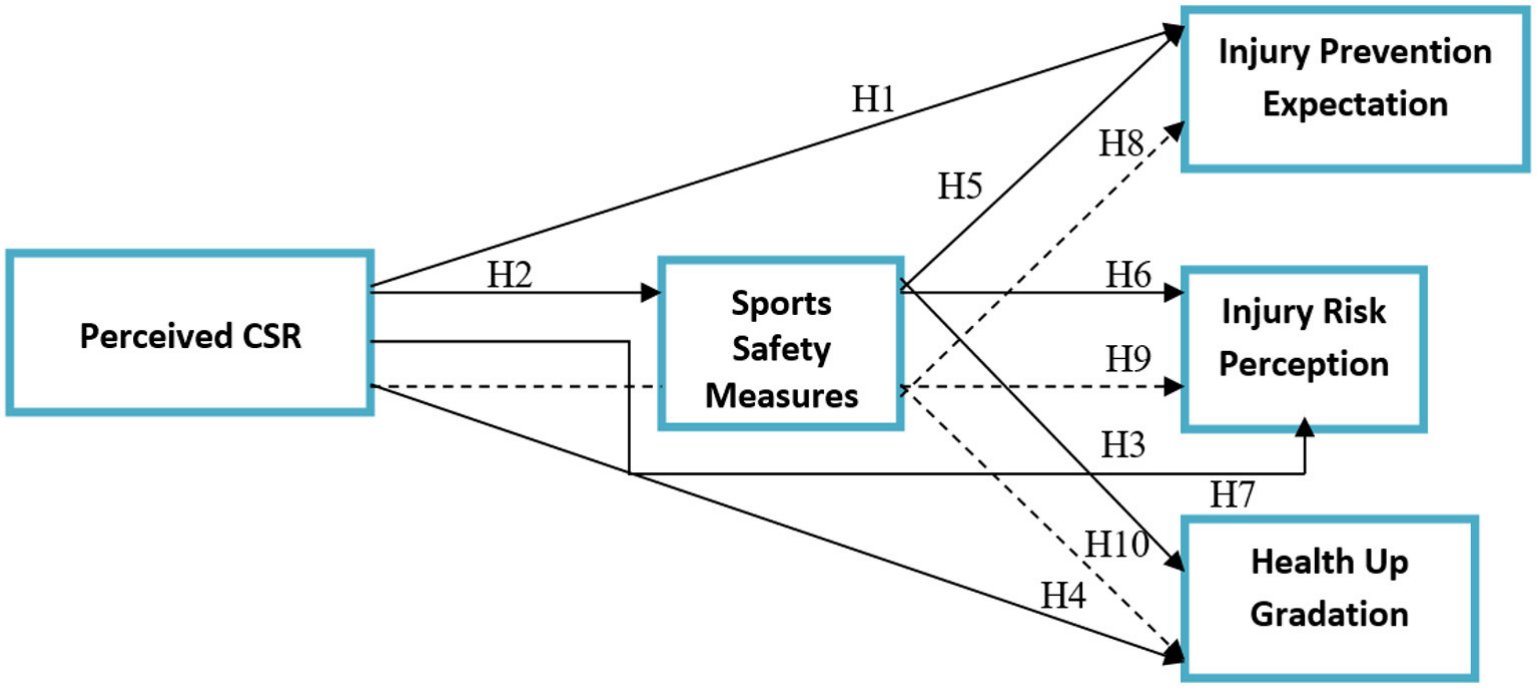
Theoretical framework. PCSR, Perceived Corporate Social Responsibility; IPE, Injury Prevention Expectation; IRP, Injury Risk Perception; HU, Health Up Gradation; SSM, Sports Safety Measures.

The theoretical framework devised from the literature review has been given in Figure 1.

## Methodology

The study intends to examine the impact of perceived corporate social responsibility on injury prevention expectation, injury risk perception, and health up-gradation. The study also analyzed the mediating effect of sports safety measures. The proposed hypotheses of the study have been developed based on the research objectives, and these hypotheses were analyzed through a deductive approach. The reliability of the data was ensured using a quantitative research design and this research design reduced any biases in the data. The data collection procedure was carried out using a self-administered survey. This survey has clarity and precision for maintaining the rationality of the data. To carry out the study, a total of 350 questionnaires were disseminated among the participants of the study. Within a time of 3 weeks, a total of 259 questionnaires were completed and properly filled out by the participants. The rest of the questionnaires were discarded during the screening procedure. After the data was collected, the data was analyzed with the aid of suitable statistical software. The target population was sportsmen of local sports bodies of China. A non-probability convenience sampling strategy was opted to select the sample from the whole target population. Nawaz et al. (2022) claimed that this method of selecting the sample takes convenient and efficient as the data is obtained from the readily available respondents. The data was obtained from individuals (unit of analysis) as the target population was sportsmen of local sports bodies of China.

### Measurement

The survey contains a 5-Point Likert scale which aided in obtaining the data from the study participants. The sections below described the measurement scale for each construct of the study.

The measurement scale of perceived corporate social responsibility had 8-items which were adopted from Zhang et al. (2021). The measurement scale of injury prevention expectation had 2-items which were adopted from Emery (2018). The measurement scale of injury risk perception had 3-items which were adopted from Emery (2018). The measurement scale of health up-gradation had 5-items which were adopted from Emery (2018). The measurement scale of sports safety measures had 9-items which were adopted from Zhang et al. (2021).

The structured Equation Modeling (SEM) technique was deployed to analyze the relationship between the study variables. As the study used this technique, the most suitable software Smart-PLS 3.3.3 opted for data analysis. Cho et al. (2020) claimed that Smart-PLS uses path models (such as measurement models and structural models) to analyze the data quickly. The models test the significance of research hypotheses along with data validity and reliability. The models also help the research to either reject or accept the proposed hypotheses by analyzing t-statistics and *p*-values (Avotra et al., 2021).

### Demographics Details

The demographic profile of the study participants has been presented in Table 1 below. These demographic factors of gender, age and education have been incorporated in the survey to get an idea about the educational background and degree of participation of each gender. Since these factors are considered important and fundamental to the sports individuals. The male and female sports persons participated in the study with 64.48% male participation and 35.52% female participation. This also showed a difference in the gender participation endorsing the findings of previous research (Burton, 2015). Age of 15 years and above is added because according the sporting rule and body for sports (in alignment with Berlin 5th Consensus on Concussion in Sport) shows that risk of injury is reduced by 60% in ages above 13 (Emery, 2018). Further, the participant taking part in the sports even formally as player (not trainer) were above age 15.The sportsmen aged between 15 to 20 years were 154 while 105 sportsmen were between 21 and 30 years. Moreover, most of the participants had bachelor's degree (65.64%), whereas master's degree holders and Ph.D. and other degree holders were 25.87 and 8.49%, respectively.

**Table 1:** Demographics analysis.

**Demographics**	**Frequency**	**Percentage**
**Gender**		
Male	167	64.48%
Female	92	35.52%
**Age (years)**		
15–20	154	59.46%
21–30	105	40.54%
**Education**		
Bachelors	170	65.64%
Masters	67	25.87%
Ph.D. and others	22	8.49%

*N = 259*.

## Data Analysis and Results

### Measurement Model

**Figure 2:**
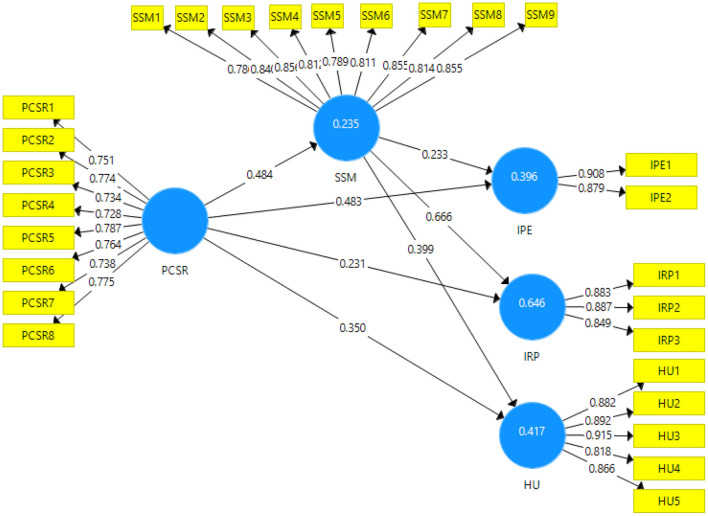
Output of measurement model. PCSR, Perceived Corporate Social Responsibility; IPE, Injury Prevention Expectation; IRP, Injury Risk Perception; HU, Health Up Gradation; SSM, Sports Safety Measures.

The output of the measurement model which presents the relationship between the independents variables with dependent variables can be seen in Figure 2.

The model assessment (direct model) is demonstrated in Table 2. The factor loadings for PCSR are between 0.728 and 0.787, for IPE are between 0.879 and 0.908, for IRP are between 0.887 and 0.894, for HU are between 0.818 and 0.915, and for SSM the factor loadings are between 0.786 and 0.856. The values obtained are above the 0.60 threshold level; therefore, all the values lie in the acceptable value range (Shah et al., 2021; Yingfei et al., 2021). Moreover, the obtained values of VIF are below 5 which the highest VIF value of 4.825, which indicates that there is no issue of collinearity in the data (Hair et al., 2017).

**Table 2:** Model assessment (Direct Model).

				**Construct reliability and validity**		
	**Factor loadings**		**VIF**	**α**	**Composite reliability**	**AVE**
	PCSR1	0.751	1.880			
	PCSR2	0.774	2.538			
Perceived corporate social responsibility	PCSR3	0.734	2.178			
	PCSR4	0.728	1.720	0.895	0.915	0.573
	PCSR5	0.787	2.996			
	PCSR6	0.764	3.220			
	PCSR7	0.738	2.793			
	PCSR8	0.775	4.791			
	IPE1	0.908	1.557			
Injury prevention expectation	IPE2	0.879	1.557	0.748	0.888	0.798
Injury risk perception	IRP1	0.883	2.075			
	IRP2	0.887	2.217	0.844	0.906	0.763
	IRP3	0.849	1.858			
	HU1	0.882	4.489			
	HU2	0.892	3.637			
Health up gradation	HU3	0.915	4.169	0.923	0.942	0.766
	HU4	0.818	2.556			
	HU5	0.866	3.131			
	SSM1	0.786	2.944			
Sports safety measures	SSM2	0.840	3.899			
	SSM3	0.856	4.102			
	SSM4	0.812	4.229	0.921	0.930	0.680
	SSM5	0.789	2.561			
	SSM6	0.811	3.136			
	SSM7	0.855	3.929			
	SSM8	0.814	4.825			
	SSM9	0.855	4.053			

*PCSR, Perceived Corporate Social Responsibility; IPE, Injury Prevention Expectation; IRP, Injury Risk Perception; HU, Health Up Gradation; SSM, Sports Safety Measures; VIF, Variance Inflation Factor; α, Cronbach Alpha; AVE, Average Variance Extracted*.

The construct reliability and validity using Cronbach alpha values, composite reliability, and AVE are also shown in Table 2. The results showed that internal consistency exists between the items of the variables as the values obtained are higher than 0.70 as PCSR (α = 0.941), IPE (α = 0.748), IRP *(*α = 0.844), HU (α = 0.923), SMM (α = 0.941) (Gualano et al., 2011). The data reliability was also analyzed using composite reliability and its value must be 0.70 (Peterson and Kim, 2013). The values obtained for composite reliability were above the acceptable value, thus the data was said to be reliable. Convergent validity was examined using AVE values and AVE should be higher than 0.50 (Archer et al., 2021). AVE values for the present study are above this threshold level (*AVE* = 0.573-*AVE* = 0.798). Therefore, indicating the presence of convergent validity.

Discriminant validity using two tests (i.e., Fornell-Larker Criterion and Heterotrait-Monotrait (HTMT) ratio) have been demonstrated in Table 3 below. The result obtained for Fornell-Larker Criterion shows that the value at the top of the column is greater than the following values on the same column (Fornell and Larcker, 1981). The discriminant validity test based on Fornell-Larker Criterion indicates that discriminant validity exists (Afthanorhan et al., 2021). The table shows that the top value of each column for heath upgradation is 0.875 and the rest of the values in the same column are lower than this. The top column value for IPE is 0.894 and the rest of the values in the same column is lower than 0.894. Table 3 also shows the values of HTMT ratio and the values are below 0.90 (*HTMT* = 0.864-*HTMT* = 0.496), thus discriminant validity based on HTMT ratio exists (Benitez et al., 2020).

**Table 3:** Discriminant validity.

**Fornell–Larcker criterion**						**Heterotrait–Monotrait ratio**					
**Constructs**	**HU**	**IPE**	**IRP**	**PCSR**	**SSM**	**Constructs**	**HU**	**IPE**	**IRP**	**PCSR**	**SSM**
**HU**	0.875					**HU**					
**IPE**	0.592	0.894				**IPE**	0.711				
**IRP**	0.626	0.627	0.873			**IRP**	0.710	0.786			
**PCSR**	0.543	0.596	0.554	0.757		**PCSR**	0.581	0.702	0.608		
**SSM**	0.568	0.467	0.778	0.484	0.825	**SSM**	0.599	0.543	0.864	0.496	

*N, 259; PCSR, Perceived Corporate Social Responsibility; IPE, Injury Prevention Expectation; IRP, Injury Risk Perception; HU, Health Up Gradation; SSM, Sports Safety Measures*.

R-square values for the variable of the study can be viewed in Table 4. Hair et al. (2014) claimed that the value of R-square near 0.50 indicates that the model is sustainable. The result obtained for R-square for health upgradation is (*R*^2^ = 0.412), for injury prevention expectation is (*R*^2^ = 0.392), for injury risk perception is (*R*^2^ = 0.643), and for sports safety measures is (*R*^2^ = 0.232).

**Table 4:** R-Square values for the variables.

	**R-Square**
HU	0.412
IPE	0.392
IRP	0.643
SSM	0.232

*N, 259; PCSR, Perceived Corporate Social Responsibility; IPE, Injury Prevention Expectation; IRP, Injury Risk Perception; HU, Health Up Gradation; SSM, Sports Safety Measures*.

According to Craney and Surles (2007), inner VIF values should be below 5 to claim that there is no issue of collinearity in the data. The results of inner VIF values explain the collinearity statistics of the data and the results for this index show that the inner VIF for HU and PCSR is (*VIF* = 1.306), for HU and SSM is (*VIF* = 1.306), for IPE and PCSR is (*VIF* = 1.306), for IPE and SSM is (*VIF* = 1.306), for IRP and PCSR is (*VIF* = 1.306), for IRP and SMM is (*VIF* = 1.306), for SMM and PCSR is (*VIF* = 1.000). As all the values are below 5, thus no collinearity issue was detected in the data set.

### Structural Model

**Figure 3:**
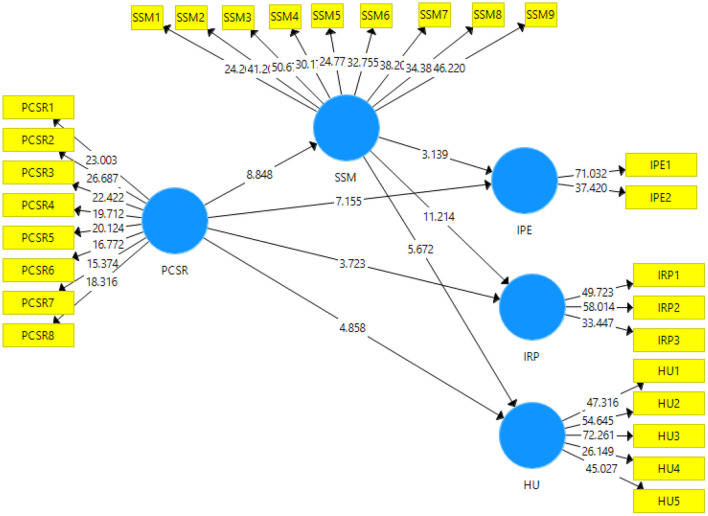
Output of structural model bootstrapping.

The value of t-statistics has been shown in Figure 3 below. This figure demonstrates the output of structural model bootstrapping. A 95% confidence interval has been taken to accept or reject the hypotheses of the study.

The results for the direct and indirect effects have been shown in Tables 5 and 6, respectively. The values of t-statistics and *p*-values determine the acceptance or rejection of the study hypotheses. A value of more than 1.96 for t-statistics signifies the acceptance of the hypotheses (Winship and Zhuo, 2020). Whereas, a value of <0.05 or with a 95% confidence interval for *p*-value suggests the acceptance of the study hypotheses (Pavlov et al., 2020). The effect size using the f-square value is also shown in Table 6. According to Cohen (1992), the value of f-square closer to 0 means the effect is weak, while, the value near to 1 means the effect is strong.

**Table 5:** Direct effects of the variable.

**Paths**	**H**	**O**	**M**	**SD**	**T-statistics**	**Effect Size (*f*^2^)**	***P*-value**	**Results**
**PCSR → IPE**	H_1_	0.483	0.492	0.067	7.155	0.296	0.000***	***Accepted***
**PCSR → SSM**	H_2_	0.484	0.487	0.055	8.848	0.306	0.000***	***Accepted***
**PCSR → IRP**	H_3_	0.231	0.235	0.062	3.723	0.115	0.000***	***Accepted***
**PCSR → HU**	H_4_	0.350	0.355	0.072	4.858	0.161	0.000***	***Accepted***
**SSM → IPE**	H_5_	0.233	0.225	0.074	3.139	0.069	0.002**	***Accepted***
**SSM → IRP**	H_6_	0.666	0.663	0.059	11.214	0.959	0.000***	***Accepted***
**SSM → HU**	H_7_	0.399	0.393	0.070	5.672	0.209	0.000***	***Accepted***

N = 259; p*** < 0.001, p** < 0.005, p* < 0.05, H, Hypothesis; O, Original Sample; M, Sample Mean; SD, Standard Deviation; SRMR= 0.096, NFI=0.669, PCSR, Perceived Corporate Social Responsibility; IPE, Injury Prevention Expectation; IRP, Injury Risk Perception; HU, Health Up Gradation; SSM, Sports Safety Measures.

**Table 6:** Indirect effects of the variable.

**Paths**	**H**	**O**	**M**	**SD**	**t-statistics**	***P*-value**	**Results**
**PCSR → SSM → IPE**	H_8_	0.113	0.109	0.037	3.059	0.002**	***Accepted***
**PCSR → SSM → IRP**	H_9_	0.322	0.322	0.043	7.516	0.000***	***Accepted***
**PCSR → SSM → HU**	H_10_	0.193	0.190	0.037	5.242	0.000***	***Accepted***

N = 259, p*** < 0.001, p** < 0.005, p* < 0.05, H, Hypothesis; O, Original Sample; M, Sample Mean; SD, Standard Deviation; PCSR, Perceived Corporate Social Responsibility; IPE, Injury Prevention Expectation; IRP, Injury Risk Perception; HU, Health Up Gradation; SSM, Sports Safety Measures.

The results for H1 to H7 (direct effects) can be seen in Table 5. The result for H1 (*t* = 7.155; *p* < 0.05) indicates the acceptance of the hypotheses, which signifies that the perceived corporate social responsibility leads to injury prevention expectations. The relationship between PCSR and IPE is weak (*f*^2^= 0.296). H2 also got accepted as (*t* = 8.848; *p* < 0.05) which means that perceived corporate social responsibility leads to sports safety measures. PCSR has a weak relationship with SSM (*f*^2^= 0.306). The outcome of H3 (*t* = 3.723; *p* < 0.05) shows that the hypothesis is accepted which means that perceived social corporate responsibility leads to injury risk perception. PCSR has a weak relationship with IRP (*f*^2^= 0.115). The result for H4 (*t* = 4.858; *p* < 0.05) indicates the acceptance of the hypotheses, which signifies that the perceived corporate social responsibility leads to health upgradation. The relationship between PCSR and HU is weak (*f*^2^= 0.161). H5 also got accepted as (*t* = 3.139; *p* < 0.05) which means that sports safety measure leads to injury prevention expectation. SSM has a very weak relationship with IPE (*f*^2^= 0.069). H6 was accepted (*t* = 11.214; *p* < 0.05) which means that sports safety measure leads to injury risk perception. SMM is very strongly related to IRP (*f*^2^= 0.959). Moreover, H7 proposed that sports safety measure leads to health upgradation, and this hypothesis was accepted (*t* = 5.672; *p* < 0.05). The relationship between SSM and HU is weak (*f*^2^ = 0.209).

The results for the indirect effects (H8 to H10) are demonstrated in Table 6. H8 got accepted as (*t* = 3.059; *p* < 0.05) which means that sports safety measure mediates the relationship between perceived corporate social responsibility and injury prevention expectation. The result for H9 (*t* = 7.516; *p* < 0.05) indicates the acceptance of the hypotheses, which signifies that the sports safety measure mediates the relationship between perceived corporate social responsibility and injury risk perception. H10 also got accepted as (*t* = 5.242; *p* < 0.05) which means that sports safety measure mediates the relationship between perceived corporate social responsibility and health upgradation.

## Discussion

This research tries to find out the possible associations between different organizational features of sports and sport-related matters during the late era of COVID-19.This research tries to find out direct as well as indirect associations which aid in boosting relationships. The duration of the pandemic affected the people in many ways which influenced their daily habits and routines. The people were confined to their homes and physical activities got limited due to the spread of viruses. This all continued for almost 2 years. Sports activities started to revamp in the late era of this pandemic and bear along with some cautions and preventive measures. As with many physical activities, there arose a concern about safety in sports activities as well. Although sports safety was already in consideration with a number of sports activities, but this pandemic put more strain on the sports activities when it came to the spread of the virus.

Most of the countries restarted their sports activities with certain preventive measures in practice such as distancing, sanitization, protective equipment and secure bubbles (Woods et al., 2020). This research contributes significantly to the sports literature as it tries to find the direct relationships of perceived CSR with sports safety measures, injury prevention expectations, injury risk perceptions and health up-gradation of people. The direct effects showed that perceived CSR was significantly associated with sports safety measures and this injury related panorama. These results indicated that when organizations connected with sports activities at outdoors and indoors and even with the sports equipment manufacturing are managed responsibly keeping in view the safety of people then it could lead to practices which help in people's well-being. When the organizations are responsibly managed in terms of social safety and environmental safety then it leads to devise good protocols which aid in managing the safety of the participants whether they are players or spectators. This can be better understood with the phenomenon of CSR in sports organizations which has been well studied before in many dimensions. Because sports organizations have long been known for their ability to provide societal advantages such as enhanced physical health, education, and social integration, the concept of corporate social responsibility (CSR) has grown in popularity across the board (Jarvie, 2003; Myers et al., 2004; Lambourne, 2006). Sports organizations differ from one another and from other enterprises in terms of core operations, organizational structures, mindsets, and strategies, therefore CSR perception in sporting settings, particularly clubs, is vital for managers and sports management personnel (Kolyperas et al., 2015).

The results showed that such perceived CSR can influence injury prevention expectations, injury risk perception and health up-gradation. The reason behind this association is supported by the fact that people become more aware of the hazards associated with sports activities when proper utilization of CSR is prevailing in their respective organizations. Previously very few studies tried to find the association between motivation for sports and these factors including injury prevention expectation, injury risk perception and health system and also found significant results (Perera et al., 2019). The research by Perera et al. (2019) provided motivation for assessing this kind of association in current research. This notion got strong support from their study.

This research also tries to find the direct association of sports safety measures with injury prevention expectations, injury risk perceptions and health up-gradation of people involved in sports activities. The results were quite similar to the previous association between perceived CSR and these outcomes. It showed that proper safety measures provided by the sports organizations to their players and audiences lead to a better sense of injury prevention and the risks of sports-related injuries. It could also play a role in the up-gradation of the health of the people involved. Previously, in this context, research was not carried out but supportive investigations also proved possible interactions like these (Ross et al., 2021). This research also evaluated the mediating role of sports safety measures taken out by sports organizations and communities involved in sports activities. The mediation between all three indirect relationships including perceived CSR, injury prevention expectations, injury risk perception and health up-gradation showed significant and positive results. This indicated that proper provision of sports safety measures by the organizations enhances the direct relationships of perceived CSR with injury prevention expectations, injury risk perception and health up-gradation. This proved its mediating role which helps in boosting these relationships. Previously, no study has found the mediating role of sports safety measures before for the sports activities, but such physical activities have been explored as a mediator between fitness-related factors (Camiletti-Moirón et al., 2020).

## Theoretical Implications, Practical Implications, and Conclusion

The crisis of COVID-19 adversely affected the sports industry. Additionally, this health crisis influenced and amended the actions and management of the local sports bodies. However, researchers found that corporate social responsibility has a positive influence on their perceptions, expectations, and health. Therefore, the study intends to explore the impact of perceived corporate social responsibility and sports safety measures on injury prevention expectation, injury risk perception, and health up-gradation with the mediation of sports safety measures among sportsmen of local sports bodies. The outcome of the analysis showed that perceived corporate social responsibility leads to injury prevention expectation, injury risk perception, and health up-gradation. Also, the study found that sports safety measure mediates the relationship between perceived corporate social responsibility and injury prevention expectation, between perceived corporate social responsibility and injury risk perception, and between perceived corporate social responsibility and health up-gradation among sportsmen of local sports bodies.

Theoretically, present examination expanded the research paradigm related to the impact of perceived corporate social responsibility on a major global crisis (COVID-19). In the sports literature, this investigation provided an in-depth understanding of PCSR on different factors such as injury prevention expectation, injury risk perception, and health up-gradation. Corporate social responsibility practices bring strategic advantages for sportsmen. In the recent era, the influence of CSR has received interest of the scholars and practitioners for motivating the sportsmen and inducing sportsmen spirit. Moreover, the mediating effect of sports safety measures has not been explored before, therefore this study would provide a deep insight into this concept and phenomenon for the readers. Most of the studies on CSR and its effect have been investigated in the context of multinational organizations and their employees; however, very few have examined CSR in the context of sportsmen, especially in the COVID-19 crisis. Thus, this theory thoroughly added value to the sports literature.

Practically, local sports bodies should actively develop strategies to adopt and implement corporate social responsibility initiatives. Such programs lead to better sports performance of the sportsmen through injury prevention expectation, injury risk perception, and health up-gradation. The managers of the local sports bodies should engage in CSR initiatives such as environmental protection and donations. Not only this, the local bodies should encourage the sportsmen to participate in such programs and be socially responsible. In the context of global crises such as COVID-19, the local sports body managers should organize and promote socially responsible efforts such as charitable actions among the sportsmen. These actions help to support the areas that are hardest hit by the COVID-19 crisis. Additionally, the managers of local sports bodies should engage communities, local governments, and non-governmental organizations to develop response plans, strengthen prevention and safety measures, and monitor pandemic risk messages for better sports performance.

## Limitations and Recommendations

The present study has a few limitations. First, the study was conducted using a small sample size; therefore, larger sample size can provide more generalized results. Results from a smaller sample size with convenience sampling can induce some biases in the results. These results can be verified with higher sample size in the European country where sports is religiously followed. This would help in comparing the results in Asia and Europe. Moreover, the present study was conducted in China, thus future studies can be conducted in different areas and regions. Another limitation of the study is the research design of the study. The study undertook a cross-sectional study design. Future studies can use a longitudinal study design in order to examine the effect of the variables in two different time periods. The study examined the role of perceived corporate social responsibility on injury prevention expectation, injury risk perception, and health up-gradation with the mediation of sports safety measures. Future studies incorporate the dimensions of CSR in the model and can examine the effect of CSR on the sports performance of sportsmen.

## Data Availability Statement

The original contributions presented in the study are included in the article/supplementary material, further inquiries can be directed to the corresponding author.

## Ethics Statement

Ethical review and approval was not required for the study on human participants in accordance with the local legislation and institutional requirements. Written informed consent from the participants was not required to participate in this study in accordance with the national legislation and the institutional requirements.

## Author Contributions

LM and JL: data collection and data acquisition. YL, YZ, and CY: literature review and methodology. LM: conceived, designed the concept, and wrote the paper. All authors read and agreed to the published version of the manuscript.

## Conflict of Interest

The authors declare that the research was conducted in the absence of any commercial or financial relationships that could be construed as a potential conflict of interest.

## Publisher's Note

All claims expressed in this article are solely those of the authors and do not necessarily represent those of their affiliated organizations, or those of the publisher, the editors and the reviewers. Any product that may be evaluated in this article, or claim that may be made by its manufacturer, is not guaranteed or endorsed by the publisher.
